# Assessment of fracture risk in women with breast cancer using current *vs* emerging guidelines

**DOI:** 10.1038/sj.bjc.6605548

**Published:** 2010-01-19

**Authors:** P Hadji, M Ziller, U S Albert, M Kalder

**Affiliations:** 1 Department of Endocrinology, Reproductive Medicine, and Osteoporosis, Philipps-University of Marburg, Baldingerstrasse 35033, Marburg, Germany; 2 Department of Gynaecology, Gynaecological Endocrinology and Oncology, Philipps-University of Marburg, Baldingerstrasse 35033, Marburg, Germany

**Keywords:** bone mineral density (BMD), breast cancer, risk factors, fracture risk, guidelines, bone loss

## Abstract

**Background:**

Breast cancer (BC) therapies can have negative effects on bone. Current guidelines recommend antiresorptive therapy based on bone mineral density (BMD), and emerging guidelines include both clinical risk factors and BMD to assess the overall fracture risk. A retrospective, case–controlled study based on current and emerging guidelines was conducted in women with newly diagnosed BC to identify those who were at increased fracture risk based on current and emerging guidelines.

**Methods:**

Baseline characteristics, fracture risk factors, and lumbar–spine (LS) and total-hip BMD in women with BC (88 premenopausal and 402 postmenopausal) were assessed to determine who would receive bisphosphonate therapy based on current and emerging guidelines.

**Results:**

Among patients with estrogen-receptor-positive (ER^+^) BC, 18.8% of premenopausal and 36.9% of postmenopausal women were osteopenic at LS. In the postmenopausal cohort, osteoporosis was more prevalent in patients with ER^+^ *vs* ER^–^ BC. Current guidelines identified 8.9% of patients as eligible for antiresorptive therapy, clinical risk factors alone identified 6.5%, and BMD plus clinical risk factors identified 28.6%.

**Conclusions:**

In addition to fracture risk factors present at BC diagnosis, cancer therapies leading to BMD loss further increase fracture risk. Evaluating both BMD and clinical risk factors may allow more effective identification of BC patients with elevated fracture risk.

Breast cancer (BC) is the most prevalent malignancy in women worldwide, with an estimated global incidence of more than 1.3 million new cases and nearly 465 000 deaths in 2007 (Garcia *et al*, 2007). Improvements in detection of breast tumours over the last few decades have resulted in the early diagnosis of the majority of BCs, thus allowing more effective therapy (American Cancer Society, 2007). Expression of estrogen receptors (ERs) and/or progesterone receptors in tumour cells is a major determinant of systemic therapy – hormone-receptor (HR)-negative BCs are typically treated using cytotoxic chemotherapy, whereas endocrine therapy is the adjuvant therapy of choice for HR^+^ tumours (Anderson *et al*, 2002; Early Breast Cancer Trialists' Collaborative Group (EBCTCG), 2005). However, both endocrine and cytotoxic therapies have known negative effects on bone health (Pfeilschifter and Diel, 2000).

Estrogen has a key role in preserving bone health in both premenopausal and postmenopausal women. After menopause, estrogen depletion is associated with increased rates of bone turnover, decreased bone mineral density (BMD), and altered microarchitecture in trabecular bone, leading to increased risk of fragility fractures (Wehrli *et al*, 2008). In addition to natural bone loss associated with ageing, therapies for BC can impair bone health directly, or indirectly by disrupting estrogen production and signalling (Pfeilschifter and Diel, 2000). For example, ovarian suppression using agents such as goserelin in premenopausal women, or aromatase inhibitor (AI) therapy in postmenopausal women, can result in substantial bone loss (Eastell *et al*, 2008; Gnant *et al*, 2008). Adjuvant AI therapy is now the standard of care for postmenopausal women with HR^+^ BC because of its superior disease-free survival results and more favourable side-effect profile (particularly with regard to venous thrombosis and endometrial cancers) compared with tamoxifen (Howell *et al*, 2005; Thurlimann *et al*, 2005; Coombes *et al*, 2007). However, AIs vary in their ability to suppress estrogen levels *in vivo* (Geisler *et al*, 2008), and recent clinical trial data indicate that the near-complete estrogen suppression associated with letrozole treatment may further improve overall survival *vs* tamoxifen in postmenopausal women with HR^+^ BC (Mouridsen *et al*, 2009). Overall, the profound estrogen depletion achieved with AI therapy is associated with decreased BMD and increased fracture risk (Coombes *et al*, 2004; Howell *et al*, 2005; Thurlimann *et al*, 2005; Eastell *et al*, 2008). Cytotoxic chemotherapy can also impair bone health either directly, through effects on bone (e.g., methotrexate), or indirectly, through premature ovarian failure (Pfeilschifter and Diel, 2000; Shapiro *et al*, 2001). Overall, both natural and cancer treatment-induced bone loss (CTIBL) can increase fracture risk in women with BC.

Fortunately, several studies demonstrated the efficacy of antiresorptive agents such as bisphosphonates for preventing BMD loss and reducing fracture risk associated with BC therapy (Gnant *et al*, 2008; Lester *et al*, 2008; Brufsky *et al*, 2009). For example, in the ABCSG-12 study (*N*=1803) in premenopausal women receiving ovarian suppression plus endocrine therapy for early stage BC, patients receiving concomitant zoledronic acid (4 mg every 6 months) for 3 years maintained BMD during the treatment period compared with a significant BMD loss in patients who did not receive bisphosphonates (−11.3% at lumbar–spine (LS), −7.3% at hip; *P*<0.0001 for both) (Gnant *et al*, 2008). Two years after therapy cessation, patients who had received zoledronic acid experienced increases in BMD (+4.0% at LS, *P*=0.022; +3.9% at hip, *P*=0.073), but BMD remained below baseline levels in the patients who did not receive zoledronic acid (−6.3% at LS, *P*=0.001; −4.1% at hip, *P*=0.058) (Gnant *et al*, 2008). In the Z-FAST study (*N*=602) at 36 months, postmenopausal women receiving zoledronic acid (4 mg every 6 months) at the start of adjuvant AI therapy had significant increases in BMD (+3.7 at LS, +1.7% at hip; *P*<0.0001 for both) compared with significant BMD losses (−3.0% at LS, −3.6% at hip; *P*<0.0001 for both) in patients for whom zoledronic acid initiation was delayed (Brufsky *et al*, 2009). In the ARIBON study (*N*=131), postmenopausal women receiving AI therapy plus ibandronate (150 mg monthly) experienced significant increases in BMD compared with placebo (+3.0% *vs* −3.2% at LS, *P*=0.002; +0.6% *vs* −3.9% at hip, *P*=0.003) (Lester *et al*, 2008). To date, there has not been a direct comparison of bisphosphonate BMD effects in the BC setting.

Established society guidelines for managing bone health in women with BC recommend the use of BMD measurements to identify candidates for preventive bisphosphonate therapy, with additional risk factors used only to identify high-risk patients who require close follow-up with dual-energy X-ray absorptiometry (DXA) (Hillner *et al*, 2003). This essentially limits bone-protective therapy to patients with DXA confirmation of osteoporosis (i.e., *T*-score ⩽−2.5). Unfortunately, DXA scanning of all patients might not be feasible in some settings. However, additional factors, including family and personal history of fragility fractures, age, long-term medications such as corticosteroids, low body mass index (BMI), and smoking, have all been associated with an increased fracture risk that is independent of BMD (Cummings *et al*, 1995; Kanis *et al*, 2005, 2007; Siris *et al*, 2006). To place BMD and other risk factors in the context of identifying women with BC who are at increased risk of fracture and are likely to benefit most from bisphosphonate therapy, a panel of experts recently evaluated data from large clinical trials in postmenopausal women and women with BC to develop international guidelines for using clinical risk factors for fracture along with BMD measurements (when available) (Hadji *et al*, 2008). To date, these emerging international guidelines have not been validated for fracture prevention in a prospective trial. Therefore, the purposes of the present single-institution study are two-fold: first, to evaluate the baseline BMD status of premenopausal and postmenopausal women with BC and the prevalence of baseline fracture risk factors in postmenopausal women with BC; second, to use these baseline data to compare current society guidelines with the non-BMD-dependent guidance for fracture risk assessment in postmenopausal patients with BC.

## Materials and methods

### Patients

Premenopausal and postmenopausal women with BC and healthy women without BC (control group) were identified from patients treated at the gynaecological department of the Philipps-University of Marburg between 2003 and 2006. Premenopausal patients were defined as those having current menses, or estradiol levels greater than 20 pg ml^−1^ and follicle-stimulating hormone levels <15 pg ml^−1^. The two groups (*N*=1184) were matched according to the following criteria: age (±9 years), weight (±16 kg), height (±13 cm), BMI (±3 kg m^−2^), hormone replacement therapy (HRT) use (1 : 1), and smoking (1 : 1). The final analysis included 88 pairs of patients and controls in the premenopausal cohort and 402 pairs in the postmenopausal cohort. All women completed a detailed questionnaire that identified all relevant risk factors for osteoporosis. The study was conducted in accordance with the Declaration of Helsinki and in accordance with the local ethical committee of the Philipps-University of Marburg.

### BMD assessment

Bone mineral density was measured by the same operator by DXA with a Lunar Prodigy densitometer (GE/Lunar Healthcare Corporation, Madison, WI, USA) using a standard protocol for the femoral neck (FN), total hip (TH), and LS. The mean interval between BC diagnosis and baseline BMD assessment was approximately 15 days for both premenopausal and postmenopausal patients.

### Guideline comparison and estimation of fracture prevention

Recommendations for managing bone health in women with BC were obtained from the American Society of Clinical Oncology (ASCO) guidelines (Hillner *et al*, 2003), and the emerging international guidelines were obtained from an expert panel of oncologists and endocrinologists (Hadji *et al*, 2008). Baseline data from postmenopausal women with BC included in this chart review were assessed according to the ASCO and emerging international guidelines to determine which patients would be eligible to receive bisphosphonate therapy. Fracture prevention was estimated based on fracture data recorded in the National Osteoporosis Risk Assessment (NORA) study in postmenopausal women (Siris *et al*, 2004) using the treatment criteria outlined in the ASCO guidelines (*T*-score ⩽−2.5) (Hillner *et al*, 2003), and the emerging international guidelines (*T*-score <−2.0 or at least two risk factors including *T*-score <−1.5, age >65 years, low BMI (<20 kg m^−2^), family history of hip fracture, personal history of fragility fracture after 50 years of age, oral corticosteroid use >6 months, and smoking) (Hadji *et al*, 2008). For this estimation, it was assumed that patients who would have received therapy according to either of the evaluation criteria would be protected from fracture. Although this is a crude estimate, it may approximate the relative protection afforded by the ASCO and emerging international guidelines, but does not consider the relative efficacy of bisphosphonate therapy.

### Statistical analyses

Data analysis was carried out using SPSS version 12.0 to test differences in the mean values between groups using the *t*-test for parametric, the *U*-test for nonparametric, and the *χ*^2^ test for categoric variables.

## Results

### Baseline patient characteristics

Among 88 pairs of premenopausal women (88 with BC and 88 controls) and 402 pairs of postmenopausal women (402 with BC and 402 controls), baseline characteristics were well balanced (Table 1). Overall, 5.8% of premenopausal women and 41.7% of postmenopausal women had received previous HRT. No women in the control group were receiving cancer therapies, whereas BC patients were undergoing standard chemotherapy or endocrine therapies that suppressed estrogen levels (i.e., tamoxifen or an AI, such as anastrozole, letrozole, or exemestane). Endocrine treatment was recorded for 79.4% of premenopausal (tamoxifen) and 91.6% of postmenopausal patients (tamoxifen or AI). Stratification by ER status revealed ER^+^ BC in 74 and 80% of the premenopausal and postmenopausal women, respectively.

### Prevalence of osteopenia, osteoporosis, and fracture risk factors

**Figure 1:**
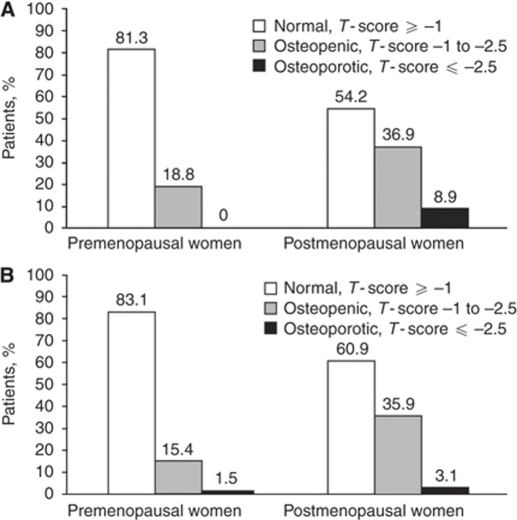
Prevalence of osteopenia and osteoporosis at (**A**) lumbar–spine and (**B**) total-hip in women with estrogen-receptor (ER)-positive breast cancer.

**Figure 2:**
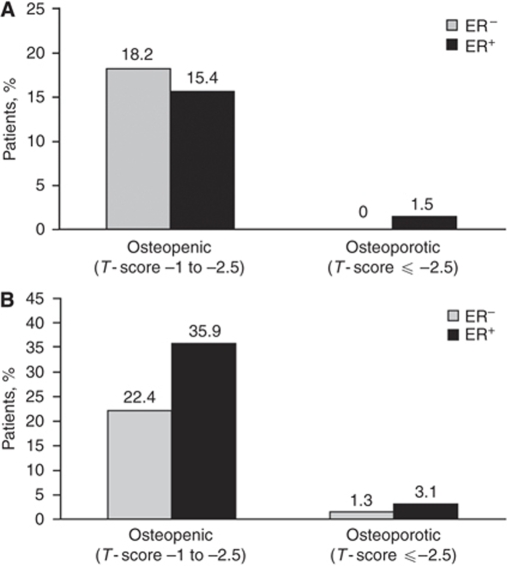
Prevalence of osteopenia and osteoporosis at the lumbar–spine in (**A**) premenopausal and (**B**) postmenopausal women with breast cancer, by estrogen-receptor (ER) status.

Among women in the control groups, the prevalence of low BMD (osteopenia or osteoporosis) was substantially higher in the postmenopausal group (⩾43%) compared with the premenopausal group (⩽25%) at all sites (FN, TH, and LS). All BC patients underwent BMD measurement shortly after diagnosis. The mean time between diagnosis and BMD measurement was 14.5 days for premenopausal and 14.8 days for postmenopausal women. Similar to patients in the control groups, the prevalence of low BMD among patients with BC was higher among postmenopausal compared with premenopausal groups. Measurements of BMD at LS (Figure 1A) and TH (Figure 1B) in patients with ER^+^ BC revealed normal BMD in more premenopausal patients compared with postmenopausal patients (>80 *vs* ∼60%). A similar BMD pattern was seen in women with ER^–^ tumours (data not shown). The overall incidence of osteopenia was 17% in premenopausal and 36% in postmenopausal patients with ER^+^ BC, whereas the corresponding osteoporosis rates were 1 and 6%, respectively. Stratification of patients by ER status revealed higher prevalence of LS osteopenia in premenopausal patients with ER^–^ *vs* ER^+^ BC (18.2 *vs* 15.4% Figure 2A), whereas LS osteopenia was more prevalent in postmenopausal patients with ER^+^ *vs* ER^–^ BC (35.9 *vs* 22.4%, respectively; Figure 2B). Moreover, LS osteoporosis was also more prevalent in postmenopausal patients with ER^+^ *vs* ER^–^ BC (3.1 *vs* 1.3% Figure 2B). The prevalence of clinical factors associated with increased fracture risk independent of BMD is presented in Table 2 (Cummings *et al*, 1995; Kanis *et al*, 2005, 2007; Siris *et al*, 2006; Hadji *et al*, 2008). Except for age (>65 years), history of smoking (∼28%), and AI therapy (∼30%), each validated fracture risk factor had low prevalence (<6%) among postmenopausal patients with ER^+^ BC; however, 44.4% of these patients had *T*-scores <−1.5.

### Assessment of the need for treatment and fracture risk using current and emerging international guidelines

**Figure 3:**
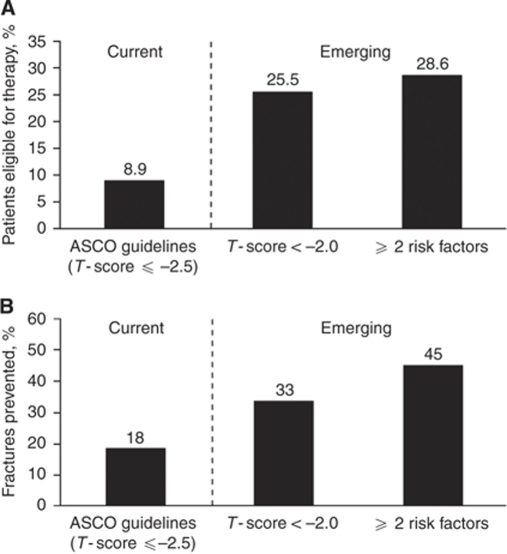
(**A**) Proportion of patients eligible for antiresorptive therapy and (**B**) estimation of the percentage of fractures prevented based on fracture-risk assessment using current (American Society of Clinical Oncology (ASCO)) and emerging international guidelines. Estimates based on fractures recorded in the National Osteoporosis Risk Assessment (NORA) study (Siris *et al*, 2004).

Current guidelines from ASCO recommend BMD screening for all women receiving AI therapy and antiresorptive agents for women with *T*-scores ⩽−2.5 (Hillner *et al*, 2003). Applying ASCO guidelines to the ER^+^ patient population in this study (i.e., the patients most likely to receive AIs), 8.9% of the postmenopausal patients would be eligible for bisphosphonate therapy (Figure 3A) (Siris *et al*, 2004). In contrast, applying the criteria from emerging international guidelines for managing AI-associated bone loss (AIBL; i.e., *T*-score < −2.0 and ⩾2 clinical risk factors including *T*-score <−1.5) (Hadji *et al*, 2008) identified more women who should receive antiresorptive therapy. Although clinical risk factors alone (i.e., not including *T*-score) identified 6.5% of postmenopausal patients with ER^+^ BC in this study as eligible for bisphosphonate treatment, 25.5% of these patients would be eligible for treatment using *T*-score <−2.0 as the sole criterion. However, selecting patients with two or more clinical risk factors (including *T*-score <−1.5) identified a somewhat larger proportion of patients (28.6%) who would be eligible for treatment (Figure 3A) (Siris *et al*, 2004). Based on NORA fracture incidence data from 170 083 women (Siris *et al*, 2006), limiting bisphosphonate treatment according to current ASCO guidelines is estimated to prevent only 18% of potential fractures, whereas treatment based on emerging international guidelines would prevent approximately 45% of potential fractures (Figure 3B) (Siris *et al*, 2004).

## Discussion

Decreased BMD and increased fracture risk are well-documented adverse events associated with BC therapies (Pfeilschifter and Diel, 2000; Hadji, 2009). Bisphosphonates have an established role in treating postmenopausal osteoporosis and have demonstrated clinical benefits for preventing bone loss during therapy for BC (Coleman, 2008; Hadji, 2009). The largest body of evidence to date is for twice-yearly intravenous zoledronic acid (4 mg), which can prevent bone loss and improve BMD in premenopausal and postmenopausal women receiving adjuvant endocrine therapy for BC (total *N*=2598) (Schenk *et al*, 2007; Brufsky *et al*, 2008; Gnant *et al*, 2008).

In Europe, current Arbeitsgemeinschaft Gynäkologische Onkologie e.V. (AGO) guidelines (www.ago-online.de) support the use of bisphosphonates to prevent and treat bone loss during adjuvant therapy for BC but do not specify a threshold for intervention, whereas the St. Gallen Expert Consensus does not recommend routine bisphosphonate use in women receiving AIs who have normal bone health (Goldhirsch *et al*, 2009). Current ASCO guidelines depend on osteoporotic BMD *T*-scores to identify patients with early BC who are eligible for bone-targeted therapy (Hillner *et al*, 2003). However, the majority of fractures occur in women who are osteopenic (BMD –1.0 to –2.5) (Siris *et al*, 2004), and treatment based on current guidelines will not prevent fractures in these women. Recent emerging international guidelines for bone health management in postmenopausal women receiving AI therapy for BC has now expanded the treatment criteria to include clinical risk factors in addition to BMD to guide therapy (Hadji *et al*, 2008).

The purpose of our study was to assess baseline characteristics in women with newly diagnosed BC and determine the proportion of patients who would receive bisphosphonate therapy based on ASCO and emerging international guidelines (Hillner *et al*, 2003; Hadji *et al*, 2008). In addition, because fracture data were unavailable for our patients, we estimated the proportion of patients in each group who would experience a fracture by comparing baseline data in our patients with fracture data from the NORA study. Results from our study indicate that the emerging international guidelines (BMD + clinical risk factors) may identify more patients likely to benefit from bisphosphonate therapy. Initiating bisphosphonate treatment according to emerging international guidelines is predicted to prevent 45% of potential fractures in postmenopausal patients *vs* 18% for treatment initiated per ASCO guidelines (Figure 3B) (Siris *et al*, 2004). These estimates are based on data from the NORA study showing that although 33% of fractures occurred in women with *T*-scores ⩽−2.0, only 18% occurred in women with osteoporotic BMD values (*T*-score ⩽−2.5), indicating that a large proportion of patients experience a fracture before developing osteoporosis (Siris *et al*, 2004). Additional data from the NORA study indicate that at least 45% of fractures occur in women with two or more fracture risk factors. It is important to note that our estimates of fracture prevention are based on fracture risk in healthy women (Siris *et al*, 2006) and might therefore underestimate the true protective effect of bisphosphonates in the BC setting.

Results from our study indicate that both premenopausal and postmenopausal women with BC may already have low BMD or develop this condition soon after BC diagnosis. Furthermore, because BMD measurement occurred within a short time from BC diagnosis (∼15 days) in this study, bone loss that would occur during BC treatment is not considered. For example, AIBL and ovarian suppression-associated bone loss are most severe within the first 3 years of beginning therapy, but remain elevated for the duration of therapy (Howell *et al*, 2005; Thurlimann *et al*, 2005; Coombes *et al*, 2007; Eastell *et al*, 2008; Gnant *et al*, 2008). Therefore, patients are likely to experience additional decreases in BMD during BC treatment, making the management of bone health an increasing concern. This is especially true for postmenopausal patients with ER^+^ BC, in whom osteopenia and osteoporosis were more prevalent at baseline. Approximately 30% (*n*=84) of postmenopausal women with ER^+^ BC were scheduled to receive AI treatment, which might further exacerbate bone loss in this group over time.

Recently, the FRAX Web-based tool (www.shef.ac.uk/FRAX/index.htm) was developed by the World Health Organization to evaluate fracture risk in healthy women based on individual patient models integrating clinical risk factors and FN BMD. These data are used to calculate the 10-year probability of hip fracture or any major osteoporotic fracture (forearm, hip, shoulder, or clinically overt in spine), with or without BMD measurements. However, because the FRAX tool does not permit including cancer therapy-associated bone loss as a variable in the fracture risk assessment, it will probably underestimate the absolute fracture risk for women with BC. Therefore, risk assessment tools such as FRAX may require specific adaptations for use in the BC population.

Our results in premenopausal and postmenopausal women with BC indicate that bone loss resulting in osteopenia and osteoporosis are relatively common in postmenopausal women with ER^+^ BC, who are likely to receive AI therapy. Because AIs are associated with increased risks of bone loss and fracture that persist for the duration of treatment, active management of bone health in this patient population is important. Using emerging international guidelines (Hadji *et al*, 2008) to initiate bisphosphonate therapy in these patients is predicted to prevent nearly half of all potential fractures. Recently developed fracture-risk evaluation tools and recommendations for monitoring bone health emphasise the importance of comprehensive fracture-risk assessment and the need for early intervention in women with BC. Future prospective studies will be needed to assess the benefits of bisphosphonate therapy for fracture prevention and to compare the relative efficacy of bisphosphonates in the adjuvant setting.

## Figures and Tables

**Table 1:** Baseline characteristics of matched patient groups

	**Premenopausal**		**Postmenopausal**	
**Characteristics**	**Healthy (*n*=88)**	**BC (*n*=88)**	**Healthy (*n*=402)**	**BC (*n*=402)**
Age (years)±s.d.	41.4±6.5	42.0±6.2	62.2±8.3	61.5±10.2
Mean body mass index (kg m^−2^)±s.d.	23.7±4.0	23.9±3.9	26.9±4.4	26.9±4.6
HRT (%)	5.8	5.8	41.7	41.7
Smoking (%)	48.8	48.8	28.3	28.3
				
*Menopausal status*				
Mean time since becoming postmenopausal (years)±s.d.	—	—	15.2±9.4	14.6±10.3
				
*Mean BMD T-score*				
TH	–0.11	–0.07	–0.68	–0.48
LS	0.26	0.03	–0.85	–0.57
				
*Endocrine treatment*^a^ (%)				
Tamoxifen	—	79.4	—	62.0
AI	—	—	—	29.6
				
Mean time from BC diagnosis to BMD (days)	—	14.5	—	14.8

Abbreviations: AI=aromatase inhibitor; BC=breast cancer; BMD=bone mineral density; HRT=hormone replacement therapy; LS=lumbar–spine; s.d.=standard deviation; TH=total-hip.

a Patients not identified as receiving endocrine treatment had hormone-receptor-negative breast cancer.

**Table 2:** Clinical risk factors for fracture and their prevalence in postmenopausal women with estrogen-receptor-positive (ER^+^) breast cancer

	**Patients**	
**Risk factors**	**Evaluable, *n***	**Risk-factor- positive, *n* (%)**
*Validated* a		
*T*-score <–1.5	322	143 (44.4)
Age >65 years	322	131 (40.7)
Low BMI (<20 kg m^−2^)	322	10 (3.1)
Family history of hip fracture	322	9 (2.8)
Personal history of fragility fracture after age 50 years	322	18 (5.6)
Oral corticosteroid use >6 months	322	2 (0.6)
Smoking	378	107 (28.3)
AI therapy	284	84 (29.6)
		
*Possible* b		
Chemotherapy	402	148 (36.8)

Abbreviations: AI=aromatase inhibitor; BMI=body mass index; ER=estrogen receptor.

a Validated in large clinical trials of healthy postmenopausal women (except AI therapy) (Cummings *et al*, 1995; Kanis *et al*, 2005, 2007; Siris *et al*, 2006; Hadji *et al*, 2008).

b Could not be validated because of insufficient trial data.
